# Mykomyringitis, or Myringitis Parasitica

**Published:** 1873-02

**Authors:** A. W. Calhoun


### MYKOMYRINGITIS, OR MYRINGITIS PARASITICA.

BY A. W. CALHOUN, M.D.

It is hoped that the above rather harsh title will not be promotive of mistrust, and the laying to one side of what follows without a perusal, but, on the other hand, that it may induce a laudable curiosity on the part of those whose attention may be attracted to it, not only to a careful reading of this short communication, but also to a still farther study and investigation of this new and interesting aural affection, which, being of recent discovery, has of late occupied no insignificant stand amongst the writings of aurists. That it has escaped detection until within the last two or three years is indeed surprising, for the same causes that generate the disease to-day have always existed, and no reason warrants a supposition that the ear is more susceptible to certain influences at present than previously, nor that the disease, if existing at a forme period, was less capable of bringing about the many troubles which call now so lustily for immediate medical treatment, and which, when rightly diagnosed and handled, yields without the slightest resistance, but unrecognized and hence mismanaged, subjects the patient to intolerable annoyance and suffering.

Interpreted in the simplest and shortest terms, the above means a parasitic inflammation of the membrana tympani. As we all know, the atmosphere around us is filled with myriads of minute vegetable particles, floating in all directions, and falling and settling in all conceivable places, and when meeting no opposition and sufficiently nourished, springing into the many parasitic forms and shapes, with some of which all of us are familiar from daily contact, but only a few of which concern us in the description of the disease in hand—myringitis parasitica, also named from the peculiar character of the vegetable parasite, mykomyringitis aspergillus. Neither does my knowledge of such things permit me, nor is it necessary or desirable in this connection, to discuss the varieties of these vegetable parasites, or their nature as to generation, growth, etc.; but for the present 'tis enough for us to know that a disease exists in the ear, called into life through the agency of these bodies, which, possessing certain characteristics, is easy of recognition, and amenable to cure by means of those remedies known as paracides.

In attending the clinics of Professors Politzer and Gruber, in this city, I have had an opportunity of observing quite a number of these cases, all of which present certain characteristics and symptoms which, when once taken well into view and study, are not afterwards easy of mistake. Almost invariably, and as amongst the first symptoms, the patient complains of what is here called "*sausen*"—a constant singing, or roaring, or drumming in the ear, which at times so increases in intensity as to become a real disease in itself. This is often a symptom or consequence of many other aural affections, accompanying not unfrequently simple acute catarrh of the eustachian tube and middle ear, and which, if any of the readers of this have at any time of their lives been possessed of, they will readily recognize as a former acquaintance, and still more readily admit as being one of the most disagreeable of all companions. But the "*sausen*" or singing is not always sufficient of itself to force the patient to seek medical advice, for he hopes and even expects that each day will bring him freedom from these distressing noises. Instead of this, they not only do not decrease, but in most cases actually increase, and in addition he finds, after a few more days, that his hearing is not as acute as formerly, and delaying still a few days longer, he discovers that, from the one or possibly both ears, he is unable to hear the ticking of his watch, except by its direct pressure upon the ear or some of the parts adjacent, and as is not unfrequently the case, he has a peculiar sensation, as if the external meatus was partially and tightly filled, or rather obstructed, by a thick mass. With all this, the individual, with but an occasional exception, is yet unwilling to class himself as amongst the diseased, and so continues the use of the many—for the most part harmless and useless—remedies recommended by each of his friends, until suddenly continuous, deep-seated and most intense, sharpest of pains, starting from the ear and running out in all directions and robbing him of sleep or rest, notify him that something must be at once done for his relief, as dangerous consequences threaten. And now he presents himself for the medical aid that he was perhaps weeks before in need of.

Pain is, to a certain extent, the preserver of most men. We neglect ourselves so long as we are at ease, even when afflicted with grave troubles, no stimulus moving us aside from the fact that we are suffering from a disease that may or may not be curable; but the advent of pain puts a new feature upon the case, exciting all the emotions conducive to the preservation of health, and determining us to immediate action in our behalf.

The patient gives usually the history and progress of his case in accordance with the symptoms above mentioned, and upon examination with the mirror, we find the membrana tympani covered with a thick, white, lifeless-looking membrane, in which we perhaps see several rugged folds, and either isolated or in groups, forming patches, we clearly discern black spots, or islands as it were, situated mostly immediately upon the membrana tympani and those parts directly around. Often has this membrane also extended out into the meatus externus, presenting the same appearances as that on the membrana tympani, viz: rough and loosened, as if separated from the deeper-laying parts, and dotted here and there with these dark patches. Occasionally, also, are these appearances accompanied by a more or less profuse secretion from the ear; or, as is frequently the case, this secretion may be the immediate trouble for which the patient presents himself, the original symptoms having in part passed away, leaving as a consequence the otorrhœa. This loosened membrane is the epidermis separated from the membrana tympani and walls of the external meatus, and these black patches are the parasitic growths, the aspergillus. By syringing the ear with a strongly-injected stream of milk-warm water, we often get the whole epidermic membrane at once, which sometimes is an exact mould of the membrana tympani and the external meatus as far outwards as the disease has extended. But we are not always so fortunate, the membrane in certain cases remaining in position notwithstanding repeated injections, or at best coming away in small pieces, and even refusing to be displaced by the forceps, except in little parcels. After this removal, we discover the parts over which the membrane had rested, viz: the membrana tympani and greater or less portion of the meatus externus, of an intensely red color, the first showing none of its former landmarks, such as processus brevis and the handle of the hammer or malleus, the light-reflecting triangular space, etc., but one solid, swollen, highly-inflamed surface, portions of the meatus being covered with excoriations produced by the disease extending on these places still deeper than the epidermis, viz: into the corium, and the membrane here, not yet entirely loosened as with the upper or epidermic layer, being torn away as it were from the deeper parts. Upon examining the membrane itself, these black spots or groups are observed, which now and then with the naked eye can be seen to be composed of hundreds if not thousands of small, separate bodies; but with an ordinary magnifying glass we get at once an idea of the form, size and number of these bodies, and are, almost without further examination, led to the diagnosis of aspergillus; but this is not positive until we place some of these more than suspicious spots under the microscope, which gives immediately an unmistakable picture, one glance at which enables us ever afterwards to easily recognize and positively diagnose the disease.

Sufficiently magnified under the microscope, each of these small parasitic particles appear as immense vegetable growths, (a sort of mushroom), consisting of a long, slender, doubly-contoured stem, occasionally giving off several branches, each increasing upon their extremities into a more or less rounded granulated head, surrounded in their turn by symmetrically-arranged, diminutive bodies, of a round form, or by such apparently pressed together, and thereby lengthened. They are classed into two different species, according to their color, viz: aspergillus flavescens and aspergillus nigricans, the latter being by far the most frequent of the two. Immediately upon removal from the ear, we can often distinguish the one from the other with the naked eye—the first presenting a yellowish-brown, and the last a smutty, very black color.

It is hardly necessary to add that the disease is found in both sexes and in all ages, two cases well demonstrative of the latter being recently treated in the clinic of Professor Politzer, viz: in a young person not yet twenty, and in an old man in his seventieth year, the latter suffering for several months, but completely cured in a few days under the treatment herein given.

The season also exercises some inexplicable influence over its appearance, it coming but seldom in summer, more frequently in the fall, and most frequently and usually in the winter. As yet, most of the cases communicated have occurred in Russia, and, as above mentioned, during the winter months; but that the disease exists in all parts of the country, and at all periods of the year, there can be no doubt.

So long as the disease is confined to the epidermis alone, but little pain is felt—or, at any rate, so slight is it as not to call for any complaint on the part of the patient. But unless at this stage the affection is rightly diagnosed and the vegetations destroyed, (which is rare, since the patient is not yet forced by the greatest severity of his trouble to consult his physician), they extend, by means of the further growth of their roots, still deeper, viz: into the corium itself—and, grasping in all directions into the already inflamed tissues, give cause to the most excrutiating, piercing pains. Knowing what a delicate structure the membrana tympani is, and how susceptible of inflammatory attacks, we can at once comprehend what an effect would be excited by the more or less rapid growth of a parasitic vegetation into its very substance, constituting in every respect a foreign body in the parts. Unfortunately, the membrana tympani once actively inflamed (myringitis), is not always so easily checked without further damage, especially when mishandled, but too often is it the case that the inflammation leads to perforation of the membrane, and, pressing still deeper, sets up a suppuration in the middle ear, accompanying which are the many evils too well known to call for explanation. Indeed, in a few of these cases, coming into the clinic on account of their otorrhœa, after cleansing the ear of the excretions and examination with the mirror, not only a perforation of the membrana tympani and suppurative inflammation of the middle ear were shown, but even the same suspicious white false membrane was again found covering the promontorium, the projecting portion of the middle ear, which without doubt had extended from the membrana tympani through the perforation into the deeper parts, preserving the same characteristics as that of the external ear.

It must not be supposed that every loose membrane upon the membrana tympani, or on the walls of the meatus externus, belong to the parasitic class or group, for often, from a slight eczema of these parts, (a not unfrequent affection), the epidermis loses its vitality, and is thrown off by the underlying layers of the cutis, presenting the apearance, and in reality forming what in common parlance is known as "*dead skin.*" Of course, upon this is nothing in particular to be found either without or with the aid of the microscope.

Amongst the other peculiarities of this disease may be mentioned the fact that, though the pain may be ever so severe, it ceases to a great extent, if not altogether, immediately upon the removal of these mushroom parasitic growths by means of the syringe or otherwise, and with their return reappears, also, the pain. This return of the parasite is no indication in all cases of insufficient treatment, for, even under the very best handling, this occasionally takes place, only to be overcome by a continuation of the treatment.

The therapeutics can be given *in toto* in a very few words. From the patient himself we not seldom learn quite a long list of remedies, with which he has already gone through, for many of these cases, for the relief of their two most prominent symptoms, viz: "*sausen,*" or singing and pain, have traveled around from point to point, consulting innumerable doctors, remaining long enough under the charge of each to be dissatisfied with their progress towards recovery, and then off to another, who very probably carries him through the same routine as he has gone through with on former and repeated occasions. Such a history I have over and over again heard from patients thus suffering, which is either the result of the physician not recognizing, and hence not properly treating the disease, (or if so, by mere accident), or, if rightly diagnosed, the suitable paracides not being used, or at any rate not being kept in use long enough, upon which the disease returns in a few days, if it had already ceased at all. All varieties of medicaments have been adopted to destroy these vegetations, such as preparations of zinc, iron, copper, silver, lead, mercury, and numbers of others, but none seem to have the effect of thoroughly destroying them. More recently, however, the remedy has been found, upon whose destructive powers we can place all confidence, viz: *the pure undiluted alcohol,* the *spiritus vini rect.* This answers every purpose so admirably well that it is now the first and only means resorted to in the treatment of the disease. In the treatment of chronic otorrhœa, it has long been successfully used by Dr. Weber, of Berlin, who first recommended its adoption by the profession in the treatment of this and other aural affections, and with what flattering results most aurists can testify.

The ear is cleansed as nearly as possible of the loosened membranes of epidermis by the use of the syringe, and forceps if necessary, and the head being turned to one side, the entire meatus externus is filled with the alcohol in a milk-warm state. This is to be retained in the ear ten or fifteen minutes, or, if the person is very sensitive, as long as he can bear it, the meatus being then emptied and a bit of *charpie* or cotton inserted to protect the parts from outside influences. This process is to be repeated three times daily till the disease has completely disappeared, and afterwards once or twice daily, according to discretion. In women and children, who are more susceptible of pain, the alcohol can, if essential, be in the beginning of the treatment diluted with water so as to give it half its original strength, and gradually lessening the portion of water, reach in a few days the use of the pure spiritis. But in most cases the full strength can be at once employed, and no fear of any important pain need be felt, as the patient experiences only for a moment an unpleasant, slightly burning sensation, which soon gives place to a feeling of an agreeable coolness in the ear. It is somewhat queer that such delicate structures as those of the external meatus, membrana tympani and internal ear, (for this, too, is often laid bare by the perforation of the membrana tympani), extremely sensitive organs even in a healthy or normal condition, can, when intensely inflamed, so well bear the use of such a powerful remedy; but it is nevertheless a fact, needing only a sufficient trial to convince the unbelieving.

It must not be imagined that a few applications of the alcohol are always sufficient to put an end to the existence of these parasitic growths, for this is frequently far from being the case, and is too often the cause of the disease continuing even after the use of this, the best remedy. The affection is very prone to a return, hence must the alcohol be vigorously applied for at least the first two weeks or more, and once a day for some time afterwards. This insures a cure, for it is impossible for the disease to return during the use of the alcohol as above recommended. A return is indicated by the reappearance of the white membrane upon one or more spots, and must be promptly met by the same means. Any excoriated places upon any part of the external meatus or membrana tympani heal quite readily by slight granulations even while the alcohol is being continued. Should otorrhœa have existed, it also rapidly disappears under this treatment, the accompanying perforation of the membrana tympani filling in a short time with cicatrices. By this simple means, a troublesome and often very painful affection can be quickly cured, and it is urged that a trial may be given it, should the disease come under the treatment of any who may chance to read the above.

VIENNA, AUSTRIA, *December* 19, 1872.

---

SUPPOSITORY FOR VAGINISMUS.—For hyperesthesia of the vulva and vagina, and for kindred affections, a French practitioner, writing to the *Gazette des Hopitaux*, strongly recommends a suppository composed as follows, to be introduced in the vagina every night: Cacao butter, thirty grains; bromide of potassium, fifteen grains; extract of belladonna, five grains.—*Pacific Medical and Surgical Journal.*

